# Pseudarthrosis after lumbar spinal fusion: the role of ^18^F-fluoride PET/CT

**DOI:** 10.1007/s00259-015-3154-y

**Published:** 2015-08-21

**Authors:** Marloes Peters, Paul Willems, Rene Weijers, Roel Wierts, Liesbeth Jutten, Christian Urbach, Chris Arts, Lodewijk van Rhijn, Boudewijn Brans

**Affiliations:** Department of Orthopedic Surgery, Maastricht University Medical Center, Postbox 5800, 6202 AZ Maastricht, The Netherlands; Radiology /Nuclear Medicine, Maastricht University Medical Center, Maastricht, The Netherlands

**Keywords:** ^18^F-Fluoride PET/CT, Bone metabolism, Interbody fusion, Low back pain, Lumbar spine, Pseudarthrosis

## Abstract

**Purpose:**

Painful pseudarthrosis is one of the most important indications for (revision) surgery after spinal fusion procedures. If pseudarthrosis is the source of recurrent pain it may require revision surgery. It is therefore of great clinical importance to ascertain if it is the source of such pain. The correlation between findings on conventional imaging (plain radiography and CT) and clinical well-being has been shown to be moderate. The goal of this study was to determine the possible role of ^18^F-fluoride PET in patients after lumbar spinal interbody fusion by investigating the relationship between PET/CT findings and clinical function and pain.

**Methods:**

A cohort of 36 patients was retrospectively included in the study after ^18^F-fluoride PET/CT for either persistent or recurrent low back pain (18 patients) or during routine postoperative investigation (18 patients) between 9 and 76 months and 11 and 14 months after posterior lumbar interbody fusion, respectively. Sixty minutes after intravenous injection of 156 – 263 MBq (mean 199 MBq, median 196 MBq) ^18^F-fluoride, PET and CT images were acquired using an integrated PET/CT scanner, followed by a diagnostic CT scan. Two observers independently scored the images. The number of bony bridges between vertebrae was scored on the CT images to quantify interbody fusion (0, 1 or 2). Vertebral endplate and intervertebral disc space uptake were evaluated visually as well as semiquantitatively following ^18^F-fluoride PET. Findings on PET and CT were correlated with clinical wellbeing as measured by validated questionnaires concerning general daily functioning (Oswestry Disability Index), pain (visual analogue scale) and general health status (EuroQol). Patients were divided into three categories based on these questionnaire scores.

**Results:**

No correlation was found between symptom severity and fusion status. However, ^18^F-fluoride activity in the vertebral endplates was significantly higher in patients in the lowest Oswestry Disability Index category (i.e. with the worst clinical performance) than in patients in higher categories (*p* = 0.01 between categories 1 and 2 and 1 and 3). The visual analogue scale and EuroQol results were similar although less pronounced, with only SUV_max_ between category 1 and 2 being significantly different (*p* = 0.04).

**Conclusion:**

We hypothesize that ^18^F-fluoride PET/CT may be able to provide support for the diagnosis of painful pseudarthrosis and could serve as a tool to discriminate between symptomatic and asymptomatic pseudarthrosis for revision surgery, as CT defines the consolidation status and PET pinpoints the ‘stress reaction’ at the vertebral endplates which significantly correlates with Oswestry Disability Index score.

## Introduction

Low back pain (LBP) is a major global health and economic problem [1], with a 1-year prevalence ranging from 22 % to 65 % and life-time prevalence of up to 84 % [2]. The direct costs in The Netherlands (15 million inhabitants), including patient care, medical procedures and medication, were estimated to be 474 million Euros in 2007. However, the yearly indirect costs caused by absence from work and early retirement in The Netherlands are much larger, i.e. 3.1 billion euros in 2007 [3].

 LBP is mainly caused by degenerative spinal disorders, such as spondylolisthesis, degenerative scoliosis, degenerative disc disease, and recurrent disc herniations [4, 5]. If conservative measures, such as intensive exercise therapy, pain medication or brace immobilization fail, operative intervention is considered. Spinal fusion is a surgical procedure that aims to eliminate painful intervertebral motion by rigid fixation using metal implants and bone grafts, to create a definitive bony fusion. One of the most used spinal fusion techniques is posterior lumbar interbody fusion (PLIF) which is characterized by a posterior surgical approach. Although multiple improvements in the surgical technique have led to higher fusion rates, failed back surgery syndrome remains a substantial problem [6]. Pseudarthrosis is defined as the absence of solid bony fusion 1 year after the operation, and occurs in at least 15 % of primary lumbar fusions [7, 8]. Pseudarthrosis is typically associated with LBP or radicular pain as a result of continued motion [6, 7]. However, pseudarthrosis may also be asymptomatic or symptoms may be atypical in a significant proportion of patients. Persistent or recurrent pain is one of the most important determinants in the decision to perform spinal revision surgery [9]. Therefore, it is essential to objectively relate pain symptoms to the degree of pseudarthrosis in order to justify revision surgery of the vertebral segments [10].

Surgical exploration is currently the gold standard for the detection of pseudarthrosis, but is highly invasive and recommended only in patients with a high suspicion of pseudarthrosis or hardware failure [6, 7, 11–13]. CT is a powerful imaging modality that allows the detection of well-established bony bridges between vertebrae. However, CT is of limited value for the diagnosis of an evolving early-stage pseudarthrosis in patients with symptoms early after surgery. Carreon et al. compared CT data with revision surgical findings in 163 patients with posterolateral fusion, and found that the diagnosis of bilateral fusion as determined using thin-slice CT was confirmed upon exploration in 96 % of the patients, indicating a high negative predictive value for pseudarthrosis. However, the absence of fusion on one or both sides on CT was a poor predictor of pseudarthrosis upon surgical exploration (low positive predictive value) [12]. Moreover, the association between CT findings and clinical symptoms is moderate [7, 14]. Nuclear bone scanning may provide earlier functional diagnosis, and can be performed using ^99m^Tc-labelled diphosphonates, suitable for SPECT/CT, or ^18^F-fluoride PET. Both tracers have similar uptake mechanisms in newly formed bone or osteoid and are therefore indicators of osteoblastic activity. However, ^18^F-fluoride PET/CT may provide images with higher resolution and sensitivity and better quantitation capabilities than ^99m^Tc SPECT/CT, that are necessary for evaluation of stress reactions and bone remodelling processes in the spine.

The hypothesis of this study was that ^18^F-fluoride PET could play a role in objectively relating clinical symptoms to pseudarthrosis, as radiopharmaceutical activity functionally correlates best with disease activity. Therefore, ^18^F-fluoride PET may help discriminate between symptomatic and asymptomatic pseudarthrosis and serve as an indicator for revision surgery. To this end, we investigated the relationship between ^18^F-fluoride PET/CT and clinical symptoms after PLIF, not only in patients with persistent or recurrent back pain, but also in patients with minor or no pain.

## Materials and methods

### Patients

A total of 36 patients who had undergone PLIF were retrospectively included in the present analysis between June 2008 and October 2014. Of these patients, 18 suffered from persistent or recurrent LBP without an apparent cause on conventional scans and diagnostics (‘persistent pain’ group) at a variable time-point after operation (range 9 to 76 months, mean 24.9 months, median 23 months). The other 18 patients were recruited as a consecutive cohort during routine clinical and radiological (plain radiography and CT) investigation 1 year after PLIF (‘postoperative’ group; range 11 to 14 months, mean 12.4 months, median 12 months). Three patients had undergone PLIF surgery at two levels. Therefore, the total number of operated levels to be analysed was 39. Operated levels were L3–L4 (*n* = 3), L4–L5 (*n* = 15) and L5–S1 (*n* = 21).

In part, this is an extension of a previous pilot study already published [15].

### Posterior lumbar interbody fusion: surgical technique

Under general anaesthesia and in a prone position, the vertebral arches of the intended levels were identified under fluoroscopic control and exposed using an open posterior lumbar approach. Nerve roots were decompressed by laminectomy and the intervertebral disc was excised. After thorough abrasion of the endplates, two 10 – 12-mm intervertebral cages (Capstone® PEEK; Medtronic, Memphis, TN), were filled with autologous bone from the vertebral lamina and inserted into the disc space. The remaining disc space was packed with additional autologous bone chips from the removed lamina. Next, the upper and lower vertebrae were fixed using four transpedicular screws connected to titanium rods (CD Legacy®; Medtronic) for primary stabilization.

### ^18^F-Fluoride PET/CT scan acquisition

Sixty minutes after intravenous injection of 156 – 263 MBq (mean 199 MBq, median 196 MBq) ^18^F-fluoride, PET and CT images were acquired with an integrated PET/CT scanner (Gemini TF PET/CT; Philips, The Netherlands). After a low-dose CT acquisition (120 kV, 30 mAs, slice thickness 4 mm) for attenuation correction, a PET scan was performed in three-dimensional mode, acquiring two 5-min bed positions covering the lumbosacral spine. This was immediately followed by a diagnostic CT scan without contrast enhancement (64-slice helical, 120 kV, 250 mAs, slice thickness 1 mm with increment of 0.8 mm) of the fusion region. Standard filtered back projection CT reconstruction was performed. PET image reconstruction included both non-attenuated and CT-based attenuated data, using time-of-flight technology. Images were viewed and postprocessed using clinical software (EBW; Philips, the Netherlands), and further analysed using dedicated research software (PMOD 3.0, PMOD Technologies Ltd, Zürich).

### ^18^F-Fluoride PET/CT data analysis

**Fig. 1:**
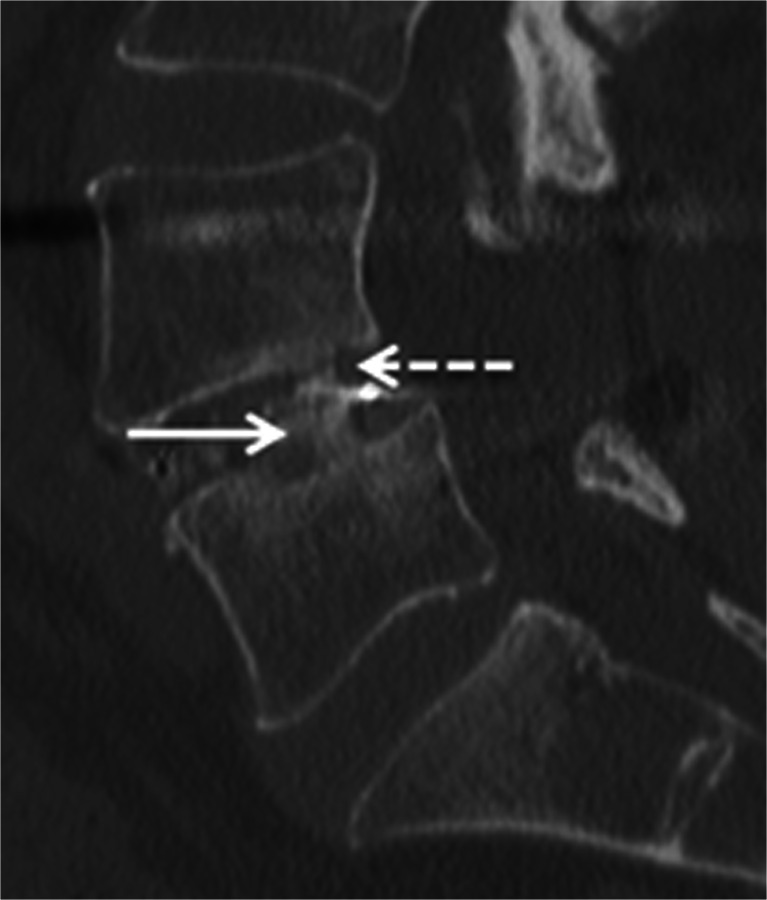
Intervertebral bony fusion. Example of a bony bridge between the cage and the lower vertebra (*closed arrow*), but not between the cage and the upper vertebra (*dotted arrow*)

**Fig. 2:**
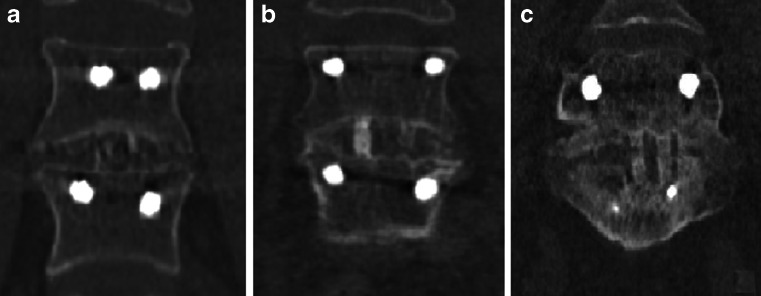
Examples of CT fusion scores 0 (**a**), 1 (**b**) and 2 (**c**)

**Fig. 3:**
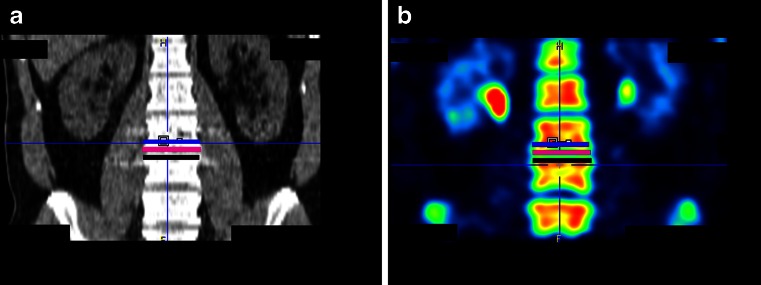
^18^F-Fluoride PET analysis of the segment operated upon. Coronal CT and PET images of the lumbar spine after posterior lumbar interbody fusion, in which three VOIs were drawn in the segment operated upon: the lower endplate of the cranial vertebra (*blue*), the intervertebral disc space (*pink*) and the upper endplate of the caudal vertebra (*black*)

The ^18^F-fluoride PET/CT scans were evaluated by two independent blinded observers (M.P., B.B.) who determined a volume of interest (VOI) for ^18^F-fluoride uptake calculation and scored bony bridging based on the standard diagnostic CT scans. Afterwards, discrepancies between the observers were resolved by consensus. Scoring was verified by an orthopaedic surgeon (P.W.) and a musculoskeletal radiologist (R.W.). Interbody fusion between the upper and lower vertebrae was classified on the diagnostic CT scan as: the presence of a bony bridge (Fig. 1) on both sides either within or around the cages (score 2); the presence of a bony bridge on one side within or around a cage, right or left (score 1); or no bridging (score 0). Examples of these fusion scores are shown in Fig. 2. On each low-dose CT scan, three ellipsoid VOIs were manually drawn following the contours of the vertebrae (slice thickness 4 mm, short axis range 40 – 50 mm, long axis range 55 – 65 mm), including the intervertebral disc space and upper and lower endplates of the segment operated upon (Fig. 3a). These VOIs were then transferred to the coregistered attenuation-corrected PET image (Fig. 3b), and in each of these VOIs, the SUV_max_ was determined, i.e. activity in the upper, lower endplates and the intervertebral disc space (SUV_max_U, SUV_max_L and SUV_max_D, respectively), as well as the ratios of the upper and lower endplate activities to the intervertebral disc space activity (SUV_ratio_U and SUV_ratio_L, respectively).

### Patient-reported outcome measures

Clinical wellbeing of the patients was evaluated using a set of validated questionnaires:

1. The Oswestry Disability Index (ODI) is the most commonly used outcome measure for LBP and disability [16], and assesses ten general daily tasks in relation to back-related function, each with six possible answers [17].
2. Pain in the back and/or legs is reliably quantified using a visual analogue scale (VAS) [9]. Patients were asked to express the amount of pain in their back, right leg and left leg separately on three scales ranging from 0 (no pain) to 100 (worst pain).
3. The EuroQol-5D (EQ-5D) is the most commonly used instrument for measuring health-related quality of life in The Netherlands [18]. The questionnaire consists of five questions each with three possible answers, each question representing a domain (i.e. mobility, self-care, usual activities, pain/discomfort and anxiety/depression). The EQ-5D index score was calculated based on a Dutch value set, representative of the Dutch population with regard to age and gender.

To facilitate further analysis of the data, each questionnaire score was linearly rescaled on a scale of 0 to 100, with 0 the worst possible score and 100 the best possible score. Patients were divided into categories based on their questionnaire score: patients with scores 0 – 40 were placed in category 1 (worst category), scores 40 – 60 in category 2 (intermediate category), and scores 60 – 100 in category 3 (best category).

### Statistical analysis

Statistical evaluation was performed using IBM SPSS Statistics for Windows, version 20.0 (IBM Corporation, Armonk, NY). To test the data for normality of distribution, the Shapiro-Wilk test was used. If data were normally distributed, an independent *t* test was used to test whether two samples originated from the same distribution. If data were not normally distributed, the Mann-Whitney *U* test was used. *P* values smaller than or equal to 0.05 were considered to indicate statistically significant differences.

## Results

Table 1 shows the distribution of SUV, CT and questionnaire scores. As can be seen, a full range of symptomatic patients as well as asymptomatic patients were included. As expected, patients who presented during follow-up with persistent back pain showed worse questionnaire scores than patients routinely scanned postoperatively. For example, regarding back-related function (ODI), 67 % of patients in the persistent pain group fell into the intermediate or worst symptom category 1 or 2, while only 11 % in the postoperative group were in these categories. Importantly, there was also high similarity between the questionnaire scores: a low or high ODI score consistently corresponded to a low or high VAS score and a low or high EQ-5D score, with *R* values of 0.9, 0.7 and 0.6 between ODI and VAS, ODI and EQ-5D and VAS and EQ-5D, respectively.

**Table 1:** Key findings

	Persistent pain group (*n* = 18)	Postoperative group (*n* = 18)
Interval primary PLIF surgery – PET/CT (months), mean (range)	24.9 (9 – 76)	12.4 (11 – 14)
Imaging findings		
PET, mean (standard deviation)		
SUV_max_ upper endplate	14.3 (4.4)	14.9 (3.9)
SUV_max_ intervertebral disc	12.5 (4.5)	15.0 (6.5)
SUV_max_ lower endplate	14.3 (5.3)	14.0 (3.6)
SUV_ratio_ upper endplate	1.2 (0.4)	1.0 (0.3)
SUV_ratio_ lower endplate	1.2 (0.5)	1.0 (0.3)
CT fusion score (*n* ^a^)		
0	7	2
1	3	9
2	12	9
Clinical findings, mean (standard deviation)		
ODI	49.9 (22.6)	82.6 (12.5)
VAS	50.7 (23.6)	81.2 (16.2)
EQ-5D	62.0 (23.8)	83.4 (14.0)

^a^Number of levels

**Fig. 4:**
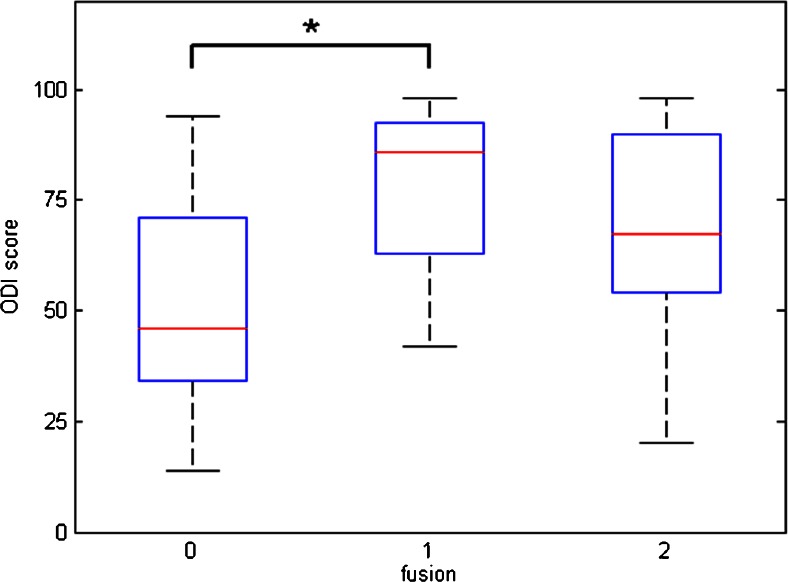
Relationship between fusion on CT (score 0, 1 or 2) and ODI score (0 – 100). Only the difference between CT scores 0 and 1 was statistically significant (*p < 0.05)

Fig. 4 shows the relationship between the intervertebral fusion score on CT and the ODI scores. ODI scores were 53.1 ± 25.3 for fusion score 0, 78.6 ± 18.0 for score 1, and 66.0 ± 25.4 for score 2 (mean ± standard deviation). Only the difference in ODI score between fusion score 0 and 1 was statistically significant (*p* = 0.017; *p* = 0.226 between scores 0 and 2, and *p* = 0.163 between scores 1 and 2). Therefore, no consistent correlation could be observed between pain severity and CT fusion score. For VAS and EQ-5D similar statistically significant results were obtained, with only statistically significant differences between groups 0 and 1 (*p* = 0.03 for both VAS and EQ-5D).

**Fig. 5:**
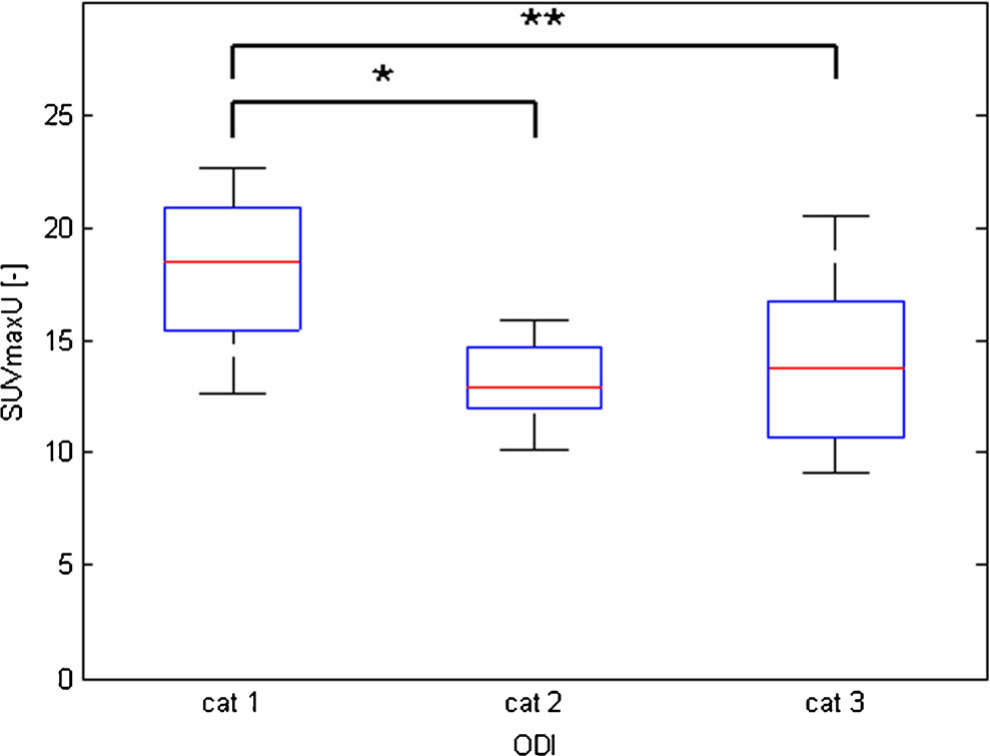
Relationship between ODI categories and SUV_max_ endplate activity on PET. SUV_max_U was significantly higher in patients in ODI category 1 (the worst clinical performance), as compared to the other categories (*p < 0.05, **p < 0.01)

Regarding PET scan values, Fig. 5 shows the relationship between the ODI category and PET endplate SUV_max_. Upper endplate SUV_max_U values were 18.1 ± 3.8 for ODI category 1, 13.2 ± 1.9 for category 2, and 13.7 ± 3.6 for category 3. The activity of the upper vertebral endplate in the entire study population was significantly higher in patients with the lowest ODI category (with the worst clinical performance) than in those with other ODI categories (*p* = 0.02 between ODI categories 1 and 2, *p* = 0.01 between 1 and 3, and *p* = 0.7 between 2 and 3). Lower endplate SUV_max_L values were 17.1 ± 5.9 for ODI category 1, 13.5 ± 2.1 for category 2, and 13.7 ± 3.6 for category 3. For the lower endplate, similar statistically significant results were found (*p* = 0.04 between ODI categories 1 and 2, *p* = 0.05 between 1 and 3, and *p* = 0.9 between 2 and 3). Intervertebral disc space SUV_max_D values were 16.8 ± 5.0 for ODI category 1, 13.6 ± 2.2 for category 2, and 12.3 ± 3.2 for category 3; there were no statistically significant differences between categories (*p* = 0.06 between ODI categories 1 and 2, *p* = 0.22 between 1 and 3, and *p* = 0.16 between 2 and 3). For VAS and EQ-5D, the results were less clear, with only SUV_max_U scores being significantly different between categories 1 and 2 (*p* = 0.04).

SUV_ratio_U was 1.2 ± 0.5 for ODI category 1, 1.0 ± 0.2 for category 2, and 1.2 ± 0.4 for category 3; there were no statistically significant differences between categories. For SUV_ratio_L, similar statistically significant results were found. For VAS and EQ-5D similar statistically significant results were also found. Thus, inclusion of the activity of the intervertebral fusion area in the PET endplate activity calculation did not result in a better correlation with symptoms.

**Fig. 6:**
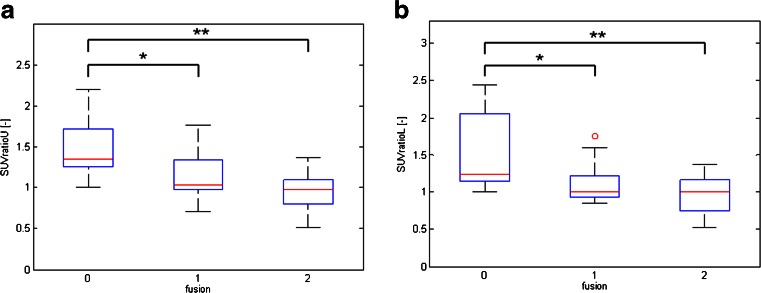
Relationship between CT fusion scores and SUV_max_ ratios. SUV_ratio_U and SUV_ratio_L in patients with pseudarthrosis (CT fusion score 0) were significantly higher than in patients with score 1 or 2 (*p < 0.05, **p < 0.01)

Fig. 6 shows the relationship between ^18^F-fluoride activity on PET (i.e. SUV_ratio_U and SUV_ratio_L) and CT fusion scores. There were a significant correlation, with SUV_ratio_U values of 1.4 ± 0.4 for fusion score 0, 1.2 ± 0.3 for fusion score 1, and 1.0 ± 0.2 for fusion score 3 (*p* = 0.04 between scores 0 and 1, *p* = 0.003 between scores 0 and 2, *p* = 0.09 between scores 1 and 2). For the lower endplate similar statistically significant results were found. SUV_ratio_U values were 1.5 ± 0.6 for fusion score 0, 1.1 ± 0.3 for fusion score 1 and 1.0 ± 0.3 for fusion score 3 (*p* = 0.05 between scores 0 and 1, *p* = 0.003 between scores 0 and 2, *p* = 0.29 between scores 1 and 2). Interestingly, the PET activity ratio in patients with fusion score 1 was not significantly lower than in patients with fusion score 2.

## Discussion

This is the first study using ^18^F-fluoride PET/CT to evaluate symptomatic as well as asymptomatic patients after spinal interbody fusion surgery and to correlate the PET and CT findings with patient-reported functionality after the surgical procedure. Pain is often multifactorial in these patients and not always alleviated by surgery. Importantly, psychosocial factors can result in a refractory state, especially after many years of debilitating pain [7]. To minimize the impact of such confounding factors, patients were surveyed using multiple patient questionnaires simultaneously. While ODI is a specific questionnaire relating to LBP and physical limitation in daily life, VAS evaluates only subjective pain intensity, and EQ-5D relates to general health status and psychological wellbeing. The scores for the three questionnaires were similar indicating that that pain, functional capacity and psychological wellbeing are affected in all patients in an integrated manner. As pain is a leading determinant in the decision to perform surgery, correlation between imaging findings and objective clinical parameters as assessed by validated and specific patient-reported outcome measures is vital.

Several studies have explored the use of ^18^F-fluoride PET and CT in patients with persistent pain after a spinal fusion procedure. Gamie and El-Maghraby [19] studied a group of 67 patients, including 25 after surgery, with back pain and negative conventional imaging, and found positive areas of uptake in facet joints and/or discs in 8 of 8 patients (100 %) who had undergone lumbar fusion. Fischer et al. [20] studied a group of 20 patients with persistent pain after cervical or lumbar fusion. They showed that even 10 years after surgery, there was increased tracer uptake around 8 of 17 cages in the intervertebral disc space, suggesting increased stress or microinstability and absence or incomplete osseous fusion. PET imaging supported the diagnosis of non-union or confirmed the diagnosis of complete fusion. Quon et al. [21] performed a prospective study in a cohort of 116 patients. In 52 of these patients (45 %) clinical evaluation and CT did not conclusively indicate the appropriate management and therefore these patients underwent PET/CT. Ultimately, 15 patients underwent revision surgery on the basis of abnormal ^18^F-fluoride foci at various sites, i.e. cages, grafts, screws, rods and fixation hardware. PET/CT correctly predicted the presence of an abnormality (cage failure, screw loosening, graft fracture) requiring surgical intervention in 14 of the 15 patients. Thus, PET/CT correctly identified patients requiring surgical management. Byrnes et al. [22] recently reported clinically useful ^18^F-fluoride PET/CT findings in 49 of 58 patients (85 %) with neck pain after cervical fusion. While these studies indicate the value of ^18^F-fluoride PET in a subset of symptomatic patients, they did not include paucisymptomatic or asymptomatic patients. In view of the low specificity of symptomatology and high sensitivity of PET which carries the risk of a substantial number of false-positive findings that is undesirable for surgical management, we think inclusion of paucisymptomatic or asymptomatic patients is crucial for the implementation of this technique.

PET activity at the vertebral endplates (SUV_max_U and SUV_max_L), but not within the intervertebral disc space (SUV_max_D, SUV_ratio_U and SUV_ratio_L), was correlated with the specific LBP and disability symptoms as measured using ODI score. We have previously found [15] that increased tracer uptake in the vertebral endplates is correlated with the occurrence and magnitude of subsidence in this type of patient, indicating instability with vertebral collapse of endplates. Subsidence is defined as sinking of a fusion cage into one or both of the adjacent vertebral bodies [23]. However, the clinical relevance of subsidence remains a matter of debate since the magnitude of subsidence does not match the final clinical results [24]. Fischer et al. [20] have also interpreted persistent increased uptake above and below the cage as inactive or unsuccessful fusion due to increase stress and microinstability. We were not able to demonstrate a consistent correlation between ODI score and CT fusion score: while significantly different ODI scores were found between CT fusion scores 0 and 1, this was paradoxically not the case between CT fusion scores 0 and 2. This could have been related to the wide range of ODI scores associated with CT fusion score 2, which may have been due to other causes of pain sensation in these patients. There appears to be an inverse correlation between the CT fusion score and SUV_ratio_ (ratio between the endplate SUV and the intervertebral disc space SUV).

 In a normally evolving fusion the number of intervertebral bridges increases from CT fusion score 0 to 1 to 2 over time, while PET activity develops from highly active endplates combined with a relatively silent intervertebral disc space (SUV_ratio_ >1.0) to an even activity distribution over the two areas (SUV_ratio_ about 1.0). This is evidence for the hypothesis that the existence of bony bridges (one or two) in combination with ‘absence’ of PET uptake indicates a stable fusion (absence of pseudarthrosis), and in the case of pseudarthrosis (no detectable bony bridges on CT), PET uptake pinpoints the symptomatic pseudarthrosis. Uptake at the endplates probably reflects ongoing stress reactions, due to an unstable lumbar fusion.

This study was limited by a lack of correlation to the gold standard of surgical exploration. We found no previous studies on the PLIF procedure that investigated the correlation between the surrogate gold standard high-resolution CT findings and surgical exploration. In posterolateral fusion, CT had a very low positive predictive value, indicating that complete mineralization of bony bridges may take substantially longer to show on CT. Prospective studies are needed that will address this in conjunction with PET findings. At our institution, implant removal is not standard practice and revision surgery is only performed in a highly selected group of pseudarthrosis patients with substantial pain. Clinical follow-up can also be used as a surrogate gold standard, but a considerable number of patients and time intervals as well as repeated PET/CT scans would be needed to fully account for all influencing confounding factors and coexisting abnormalities. Therefore, the potential of the PET scan to predict therapy management could not be fully assessed in this study. However, the main goal of this study was to evaluate the relationship between active PET scan abnormalities and symptoms. Furthermore, large differences between patients in the time between fusion surgery and ^18^F-fluoride PET/CT examination were present. A similar follow-up period for all patients would have been ideal, but was not feasible within the limits of clinical practice, because symptomatic patients can present with pain at any time from months to years after PLIF. For asymptomatic patients, it is not feasible to perform PET/CT many months to years after a successful fusion procedure. Finally, another limitation of the study was the small sample size.

In conclusion, our study demonstrated that ^18^F-fluoride uptake on PET correlates better with symptomatic pseudarthrosis than the CT findings. Furthermore, an inversed relationship between PET uptake and CT score was found.
